# Leaf Trait Networks Based on Global Data: Representing Variation and Adaptation in Plants

**DOI:** 10.3389/fpls.2021.710530

**Published:** 2021-12-07

**Authors:** Ying Li, Congcong Liu, Li Xu, Mingxu Li, Jiahui Zhang, Nianpeng He

**Affiliations:** ^1^Key Laboratory of Ecosystem Network Observation and Modeling, Institute of Geographic Sciences and Natural Resources Research, Chinese Academy of Sciences, Beijing, China; ^2^College of Resources and Environment, University of Chinese Academy of Sciences, Beijing, China; ^3^Key Laboratory of Vegetation Ecology, Ministry of Education, Northeast Normal University, Changchun, China

**Keywords:** functional trait, network analysis, leaf trait network, adaptation, leaf ecnomic traits

## Abstract

The interdependence of multiple traits allows plants to perform multiple functions. Acquiring an accurate representation of the interdependence of plant traits could advance our understanding of the adaptative strategies of plants. However, few studies focus on complex relationships among multiple traits. Here, we proposed use of leaf trait networks (LTNs) to capture the complex relationships among traits, allowing us to visualize all relationships and quantify how they differ through network parameters. We established LTNs using six leaf economic traits. It showed that significant differences in LTNs of different life forms and growth forms. The trait relationships of broad-leaved trees were tighter than conifers; thus, broad-leaved trees could be more efficient than conifers. The trait relationships of shrubs were tighter than trees because shrubs require multiple traits to co-operate efficiently to perform multiple functions for thriving in limited resources. Furthermore, leaf nitrogen concentration and life span had the highest centrality in LTNs; consequently, the environmental selection of these two traits might impact the whole phenotype. In conclusion, LTNs are useful tools for identifying key traits and quantifying the interdependence of multiple traits.

## Introduction

**FIGURE 1:**
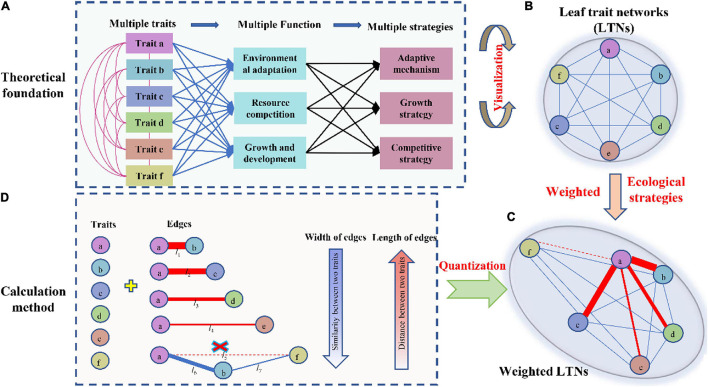
Theoretical basi s and method used to calculate leaf trait networks (LTNs). Multiple leaf traits jointly interact with each other to adapt to the environment or to optimize leaf functions. Integrative LTNs could help capture highly complex relationships among different traits and explore the underlying strategies of plants **(A,B)**. Considering that plants can adjust their relationships through strength and distance, actual LTNs are shown in panel **(C)**. LTNs can be represented as a set of nodes (circles) connected by edges (lines) **(D)**.

Plant functional traits are defined as morpho-physio-phenological traits that indirectly impact fitness *via* their effects on growth, reproduction, and survival (Violle et al., 2007; He et al., 2018; Liu et al., 2021). Functional traits are not independent of each other, and their relationships are often represented by positive and negative correlations and allometry, resulting from the different biomechanical and physiological requirements of plants (Freschet et al., 2015). Bivariate trait relationships mainly arise from three different reasons (Sack et al., 2013) (1) direct mechanistic (i.e., physiological structure function relationships), where for instance the size or number of a given structure determines the physiological output of a process; (2) optimal design, in which each trait independently contributes structurally to an overarching function (Sack and Holbrook, 2006); and (3) concerted convergence, in which each trait contributes independently to advantage in a given environment (Givnish et al., 2005). In particular, bivariate trait relationships have been tested from the species to the community level, and from the local to global scale (Bruelheide et al., 2018). However, many functional traits interact with each other and jointly optimize functioning, allowing plants to apply multiple strategies for environmental adaptation, resource competition, and development (Figure 1A; Diaz et al., 2016; La Riva et al., 2016; Bruelheide et al., 2018; Liu et al., 2019). Thus, focusing on the interdependence of multiple traits, rather than bivariate trait relationships, could provide more realistic insights into how plants adapt to their environment.

There are many ways to show the interdependence of multiple traits. Correlation matrices and heatmaps are often used to describe trait–trait interrelationships (Reich et al., 1999); however, both these approaches are limited to quantifying bivariate trait relationships. Structural equation models can be used to study the interrelationships of multiple traits (Vile et al., 2006); however, such models are often used to analyze directed relationships (e.g., cause and effect). Yet, most traits are equal in status. Principal component analysis has been used to analyze multiple traits and group them as independent units by clustering. Within this framework, ecologists strive to reduce the number of linkages among multiple traits to a few axes of variation, also termed “spectra” or “leading dimensions” (Westoby et al., 2002; Diaz et al., 2004; Laughlin, 2014). Furthermore, leaf economic traits and hydraulic traits are decoupled in tropical-subtropical forests (Li et al., 2015). However, dimensionality reduction and clustering methods only qualitatively (not quantitatively) describe the interrelationships of traits within groups and among groups. Network analysis seems to be an effective solution to this problem.

A few studies have applied network visualization to show how traits are correlated (Poorter et al., 2013, 2014; Mason and Donovan, 2015; Schneider et al., 2017). In addition to providing a tool for network visualization, trait networks also could capture variation in the interdependency of traits, allowing the important traits of plants to be distinguished using network parameters (Messier et al., 2017; Kleyer et al., 2019; He et al., 2020). Recently, Kleyer et al. (2019) used network parameters to explore the relationships among plant traits. The authors showed that stem mass and stem-specific length are “hub” traits, meaning that they are correlated with most traits.

He et al. (2020) pointed out that plant trait network is an effective method to explore the complex relationship between multiple plant traits, and made some prospects for the application of trait network. Network analysis has rigorous network parameters (Table 1), with this approach potentially providing a higher resolution of the complex relationships among multiple leaf traits.

**TABLE 1:** Key parameters of leaf trait networks (LTNs).

	Parameters	Definition	Ecological significance
Overall parameters	Edge density (*ED*)	*ED* was the ratio of the sum of actual weighted edges to the maximum possible weighted edges.	A network with higher *ED* may allow for the more efficient acquisition and mobilization of resources (as all traits are connected with all other traits)
	
	Diameter (*D*)	*D* refers to the maximum shortest path length between any two connected traits in the network.	Higher *D* represent higher independence among any plant traits
	
	Average path length (*AL*)	*AL* was the mean shortest path between all traits in the network.	Higher *AL* represent stronger interdependence among plant traits

Individual parameters	Degree (*k*)	*k* was defined as the sum of edges that connect the focal node traits to other nodes, and the number of connections and the strength of relationships influence the degree of trait.	Traits with higher *k* favor the efficient use and acquisition of resources within and across plant tissues
	
	Closeness (*C*)	*C* was defined as the reciprocal of the mean shortest path between a focal node trait and all other nodes in LTNs.	Traits with higher *C* refer to the traits closely related to other traits in the network
	
	Betweenness (*B*)	*B* was defined by the number of shortest paths going through a focal node trait.	Traits with higher B values could serve as a broker in the network

The worldwide leaf economics spectrum consists of leaf chemical, structural, and physiological traits. This spectrum was used to show that fast-growing species have higher photosynthetic rates and nitrogen concentrations than slow-growing species, which have higher leaf mass per area and higher leaf longevity (Wright et al., 2004; Wright and Suttongrier, 2012). Fives models were used to explain these patterns (two based on structural allocation, two on venation networks, and one on resource allocation to cell walls and cell contents). Each model yielded different explanations for the correlation between these functional traits (Benjamin et al., 2015). Using graph theoretic methods and structural equation modeling, Shipley et al. (2006) showed that the trade-off strategy of plants may lead to a certain quantitative relationship among leaf mass per area (LMA), photosynthetic assimilation rate (A_mass_), and leaf lifespan (LL). The origins of the bivariate trait relationships between leaf economic traits are controversial; however, their bivariate trait relationships have been recorded at multiple scales (Wright et al., 2005; Wright and Suttongrier, 2012). Because all leaf economic traits are closely correlated with photosynthesis and productivity, it is necessary to quantify variation in the interdependency of leaf economic traits.

Mediating the trade-off between cost and benefit, leaf economic traits interactively and jointly optimize photosynthesis; consequently, their complex relationships are expected to form a huge network (Figure 1B). This network is represented as a set of nodes (traits) connected by edges (bivariate trait relationships). The width and length of edges are important for network analysis (Figure 1D), and given those bivariate trait relationships between leaf economic traits are observed from local to global scales, weighted trait networks must be established (Figure 1C). This approach facilitates the accurate expression and measurement of the interdependency of leaf economic traits. Specifically, here we aimed: (1) construct leaf trait networks (LTNs) using leaf economic traits and reveal their complex relationships; (2) explore differences in the interdependence of multiple traits among different growth forms and life forms; and (3) identify the key traits among six leaf economic traits.

## Materials and Methods

Global data on six key traits of leaves were obtained from the GlopNet which was also available in the TRY Plant Trait Database^1^. It includes 2548 species of 219 families in 175 sites (Supplementary Table 1; Wright et al., 2004). Covering all major biome types, it represents a wide range of vegetation types from arctic tundra to tropical rain forest, from hot to cold desert, and from boreal forest to grassland (Wright et al., 2004). Plants are divided into different growth forms (trees, shrubs, herbs, etc.) and different life forms (coniferous and broad-leaved). These traits were leaf mass per area (LMA), photosynthetic assimilation rate (A_mass_), leaf nitrogen (N_mass_), leaf phosphorus (P_mass_), dark respiration rate (R_mass_), and leaf lifespan (LL). In practice, these traits could also be expressed based on area except for LMA and LL (including A_area_, N_area_, P_area_ and R_area_), reflecting light capture, and energy transaction for example, N_area_ = N_mas_ × LMA. Osnas et al. (2013) pointed out that both mass standardized and area standardized leaf economic spectra and LMA revealed the internal links between the physiological and ecological functions of plant traits, but these links were not consistent with the relationship simulated by the global vegetation model to solve the problem of climate change. Consequently, we conducted a parallel analysis using mass-based and area-based traits as conducted by Wright et al. (2004) (see Supplementary Tables 1, 2 for details of traits). The “area standardized” traits data of different types of plants were less, which was not enough for network analysis. Therefore, the LTNs of different growth forms and life forms were only analyzed based on “mass standardized” traits.

### Establishment of Leaf Trait Networks

Leaf trait networks are multi-dimensional networks that consist of nodes and edges. To create the correlations for leaf traits in LTNs, all leaf traits were designated as nodes, while trait–trait relationships were delineated as edges. First, the correlation coefficient matrix of leaf traits was calculated (Supplementary Figure 1). The strength of trait–trait relationships were described using absolute Pearson correlations (| r|). A threshold of marked pairwise correlations was used, with *P* < 0.05 considered a significant difference. Other relationships were set to zero, yielding the adjacency matrix *A* = [*a*_i,j_] with *a*_i,j_ ∈ [0,1]. Additional network connections between any pair of leaf traits were weighted by the absolute correlation strength (Kleyer et al., 2019). LTNs were visualized using the improved package “igraph” in R software.

The distance between trait nodes was calculated following set criteria. First, principal component analysis (Supplementary Table 3) was conducted for all six leaf traits, removing the influence of dimension and correlation between traits. The Euclidean distance between two traits was calculated as the distance between two traits in LTNs (Supplementary Figure 2).


(1) $$d_{i\,j}=\sqrt{\sum_{m=1}^{6}{\left(j_{m}-i_{m}\right)}^{2}}$$

where, *d*_ij_ is the distance between the focal node trait *v*_i_ and node trait *v*_j_. *i*_m_, and *j*_m_ are the score of traits *i* and *j* in the m^*th*^ principal component.

### Overall Parameters of Leaf Trait Networks

Three overall network parameters were considered here (Table 1): *edge density* (*ED*), *diameter* (*D*), and *average path length* (*AL*).

*ED* is the ratio of the sum of actual weighted edges to the maximum possible weighted edges. A network with a high *ED* might allow for the efficient acquisition and mobilization of resources (because all traits are associated with all other traits); however, this phenomenon might be costly for plants in terms of the establishment and maintenance of connections among traits (Flores-Moreno et al., 2019; He et al., 2020). A higher *ED* indicates closer trait relationships in LTNs.


(2) $$E\,D=\frac{1}{n\cdot \left(n-1\right)}\,\sum k_{i}$$

where, *k*_i_ is the *degree* of focal node trait *v*_i_, and n is the number of node traits.

The *path* between two nodes *i* and *j* consists of the edges to pass from node *i* to node *j*. The *path length* is the sum of edges passed. The *shortest path length d*_ij_ is the minimum of all path lengths connecting node *i* to node *j.*

*D* is the maximum *shortest path length* between any two connected node traits in the network. *AL* is the mean *shortest path length* between all node traits in the network. LTNs with higher *D* and *AL* indicate an overall higher independence among any leaf traits, with lower resource use and acquisition efficiency.


(3) $$D=max\left\{d_{i\,j}\right\}\left(i\neq j\right)$$

(4) $$A\,L=\frac{1}{n\cdot \left(n-1\right)}\,\sum i\neq j\,d_{i\,j}$$

where, *d*_ij_ is the *shortest path length* between focal node trait *v*_i_, and node trait *v*_j_, n is the number of node traits.

### Individual Parameters of Node Traits in Leaf Trait Networks

Nodes represent traits in LTNs; Two parameters can be used to quantify the connectedness of each trait, that is, the *degree* (*k*) and *closeness* (*C*); and a parameter to quantify the centrality of each trait, that is, the *betweenness* (*B*) (Table 1; He et al., 2020).

*Degree* (*k*) is the sum of edges that connect focal node traits to other nodes. Leaf traits that have a higher *k* favor the efficient use and acquisition of resources (Reich and Cornelissen, 2014), which were delineated as hub traits in LTNs.


(5) $$k_{i}=\sum j\neq i\,a_{i\,j}$$

where, *a*_ij_ is the connection (Pearson correlations | r|) between focal node trait *v*_i_ and node trait *v*_j_.

*Closeness* (*C*) is the reciprocal of the shortest mean path between a focal node trait and another node in LTNs.


(6) $$C_{i}=\frac{n-1}{\sum_{j=1}^{n-1}d_{i\,j}}\left(i\neq j\right)$$

where, *d*_ij_ is the shortest distance between focal node trait *v*_i_ and node trait *v*_j_, and n is the number of node traits in LTNs.

*Betweenness* (*B*) is the number of shortest paths passing through a focal node trait. Traits with a higher *betweenness* serve as brokers (i.e., traits with high betweenness likely coordinate several subnetworks) (Kleyer et al., 2019).


(7) $$B_{i}=\sum j\,k\,\sigma \,\left(j,i,k\right)$$

where, σ (*j*, *i*, *k*) is the number of shortest paths between focal node trait *v*_j_ and node trait *v*_k_, which crosses node *v*_i_.

### Data Analyses

The parameters of node traits and overall LTNs were calculated using the package “igraph” of R. To obtain uncertainty ranges of these parameters, we randomly resampled plant species 9999 times, and an LTN was established for each bootstrapping. Then, the “average” and “standard error” values for these parameters were calculated. Mass-based LTNs and area-based LTNs were also established. This process was applied to different growth forms (because there are few trait data of herbs, network analysis was only used for trees and shrubs) and life forms (conifer and broadleaf). Given the limited trait data, the number of species selected at random was more than three-quarters the number of specific species pools to ensure that each bootstrapping could establish an LTN.

An independent sample *t*-test was used to compare how LTNs differed among different growth forms (trees and shrubs) and plant life forms (conifer and broadleaf). One-way ANOVA was used to compare how node parameters differed with different leaf traits. Multiple paired comparisons were used to explore the paired mean difference for parameters of different forms.

Data analyses and visualization were performed using R software (version 3.3.1, R Core Team, 2016) and a web application^2^ (Ho et al., 2019). Significance was set at *P* < 0.05.

## Results

### Leaf Trait Networks for Six Leaf Traits Based on Global Data

**FIGURE 2:**
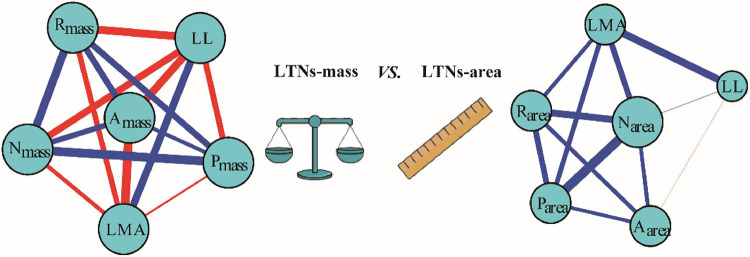
Leaf traits networks (LTNs) for six leaf traits based on global data. Red and blue edges show negative and positive correlations, respectively. The correlation strength among traits is shown by line thickness. The node size is shown as degree. Data on leaf traits were derived from the report by Wright et al. (2004).

**FIGURE 3:**
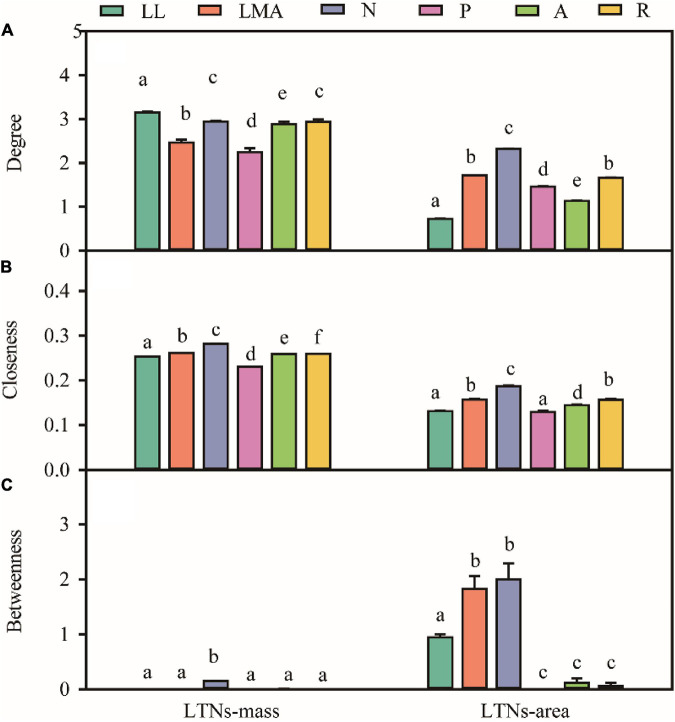
Variation in degree **(A)**, closeness **(B)**, betweenness **(C)** for different leaf trait networks (LTNs) on mass-based and area-based leaf traits. Different letters indicated the significant difference (*P* < 0.05). Error bars were represented standard error (SE).

Based on the global dataset of six leaf traits, we constructed leaf trait networks using mass (LTNs-mass) and calculated the overall parameters and individual parameters of LTNs-mass (Figure 2). *ED*, *D*, and *AL* of LTNs-mass were 0.42–0.65, 1.00–1.43, and 0.75–0.84, respectively, with average values of 0.55, 1.12, and 0.78 (Supplementary Figure 3 and Supplementary Table 4). *k*, *C*, and *B* differed significantly for all six leaf traits (*P* < 0.05 for all; Figure 3, Supplementary Tables 5–8). LL had a higher *k*, N_mass_ had higher *C*, and *B* for all six leaf traits was close to 0, except for N_mass_.

Our parallel evaluation of leaf trait networks based on area (LTNs-area) (Figure 2) demonstrated significant differences between LTNs-mass and LTNs-area. The *ED* of LTNs-area was significantly lower than LTNs-mass. The *D* and *AL* of LTNs-area were significantly higher than LTNs-mass (Supplementary Figure 3, Supplementary Table 4). Details on the LTNs-area analysis were presented in Figure 3, Supplementary Figures 3, 4, and Supplementary Tables 4–9.

### Differences to Leaf Trait Networks Among Plant Growth Forms

**FIGURE 4:**
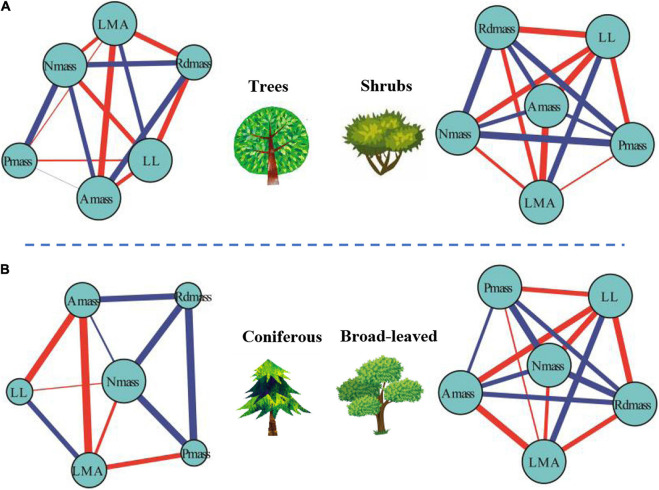
Differences of leaf traits networks (LTNs) in different plant growth forms **(A)** and plant life forms **(B)** based on global data. Red and blue edges show negative and positive correlations, respectively. The correlation strength among traits is shown by line thickness. The node size is shown as degree.

**FIGURE 5:**
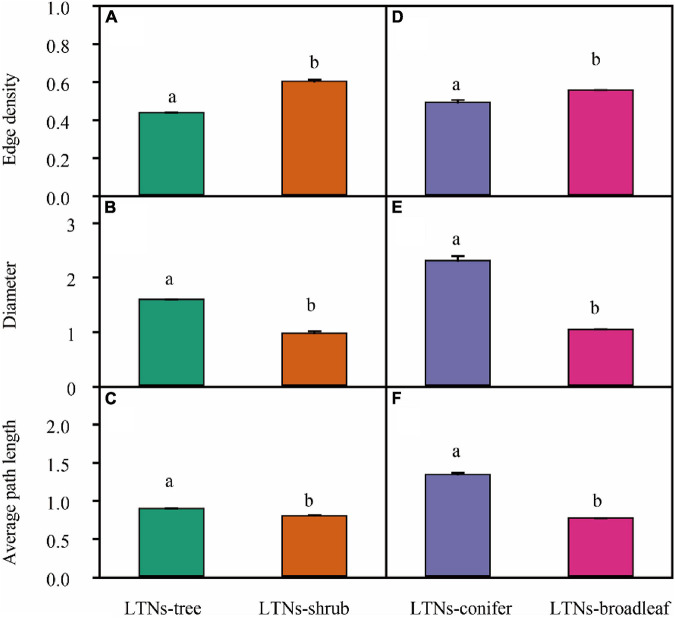
Variation in the overall parameters of leaf trait networks (LTNs) among different plant growth forms **(A–C)** and plant life forms (**D–F**). Different letters show a significant difference between two networks (*P* < 0.05). Error bars represent standard error (SE).

For mass-based leaf traits, we classified plants into different plant growth forms and explored the differences within the framework of LTNs. LTNs facilitate the visualization of complex relationships among traits, showing the interdependence of traits from different growth forms (Figure 4A). The *ED* of the leaf trait network of shrubs (LTNs-shrub) was significantly higher than that of trees (LTNs-tree) (*P* < 0.05) (Figure 5A, Supplementary Table 2). The *D* and *AL* of LTNs-tree were significantly higher than those of LTNs-shrub (*P* < 0.05 for all) (Figures 5B,C, Supplementary Table 4). The paired mean difference of *k* and *B* between LTNs-tree and LTNs-shrub was 0.83 and −0.18, respectively (*P* < 0.05 for all) (Supplementary Figures 5A,C, Supplementary Table 4). No significant difference was detected for the paired comparison of *C* and shortest path length between LTNs-shrub and LTNs-tree (Supplementary Figures 5B,D, Supplementary Table 5). N_mass_ had a higher *k* and *B* than the other five traits for LTNs-tree (*P* < 0.05 for all). Furthermore, A_mass_ had a higher *C* for LTNs-tree. For LTNs-shrub, LL had the highest *k*, while N_mass_ had the highest *C* and *B* (*P* < 0.05 for all) (Supplementary Figure 6, Supplementary Tables 6–8). Information on the shortest path lengths is presented in Supplementary Table 10.

### Differences of Leaf Trait Networks Among Plant Life Forms

The *ED* of the leaf trait network for broadleaved trees (LTNs-broadleaf) was higher than that of conifers (LTNs-conifer) (Figures 4B, 5D, Supplementary Table 4). The *D* and *AL* of LTNs-conifer were higher than those of LTNs-broadleaf (Figures 5E,F, Supplementary Table 4). The paired mean difference of *k*, *C*, and *B* between these two tree groups was 0.28 (*P* > 0.05) (Supplementary Figure 7A, Supplementary Table 5), 0.10 (*P* < 0.05) (Supplementary Figure 7A, Supplementary Table 5), and −0.73 (*P* < 0.05) (Supplementary Figure 7C, Supplementary Table 5), respectively. The paired mean difference of shortest path length between these two groups was –0.55 (*P* < 0.05) (Supplementary Figure 7D, Supplementary Table 5).

The node parameters of different LTNs were significantly different for both LTNs-conifer and LTNs-broadleaf. P_mass_ and LL had the highest *k* for LTNs-conifer and LTNs-broadleaf (*P* < 0.05 for all), respectively. N_mass_ had the highest *C* and *B* for LTNs-conifer and LTNs-broadleaf (*P* < 0.05 for all) (Supplementary Figure 8, Supplementary Tables 6–8). Information on the shortest path length was presented in Supplementary Table 11.

## Discussion

### Leaf Trait Networks Provide an Effective Approach for Exploring Complex Relationships Among Leaf Economic Traits

The interdependency of multiple traits is the basis of multiple functions. Due to environmental pressure and plant trade-off strategy, there will be a certain quantitative relationship between traits with different functions. Many relationships were integrated into leaf organs and plant level to form a complex and orderly trade-off relationship network of economic spectrum traits. Throughout the relevant studies of leaf economic spectrum (Wright et al., 2004, 2005; Shipley et al., 2006; Osnas et al., 2013), it is always inseparable from the exploration of the pattern of leaf functional traits and the trade-off relationship between them, in other words, the complex and stable “economic” strategies and relationships between functional traits, It is the basis and starting point of leaf economics spectrum research, and functional traits are the nodes of these relationship networks. Therefore, the network analysis was used to explore the complex relationships among plant traits here.

There are some minor, but fundamental differences between LTNs and traditional network analysis. For example, the microbial network is used to explore how soil microorganisms coexist, whereas leaf traits are permanent and cannot be removed from plants (Wang et al., 2018). In transportation networks, the distance between airports/railway stations is real and measurable, whereas the distance between leaf traits is difficult to quantify (Wang et al., 2011). Studies conducted within the last 10 years established the basis of the plant trait network, completing the visualization of interdependent relationships among multiple traits (Poorter et al., 2013, 2014; Sack et al., 2013; Mason and Donovan, 2015; Messier et al., 2017; Kleyer et al., 2019; He et al., 2020). However, the concept of using the parameters of a network in an LTN to quantify variation in the interdependency of multiple traits is novel and effective. Other fields of research have already developed methods to construct networks and evaluate associated parameters. For instance, a special website for network structure and parameter analysis has been developed in the field of molecular ecology (Deng et al., 2012). However, the edges of LTNs differ from those of the microbial network and transportation network. Thus, we weighted the network with the absolute value of the correlation coefficient. There is general consensus that the more similar the traits, the closer the distance between them (Kleyer et al., 2019). However, the calculation of the distance between traits is a challenge. Some scholars used unweighted approaches to construct an unweighted network that connects traits (Flores-Moreno et al., 2019). Studies within the last 5 years used the reciprocal of the correlation coefficient as a proxy for the distance between multiple traits (Kleyer et al., 2019); Here, we explored using the Euclidean distance under the principal component of traits as the distance between traits. Within this framework, we calculated the relevant network parameters. We recommend that this approach may be widely implemented in future studies investigating plant trait networks.

Many studies compared and analyzed the indication intensity of two types of standardized traits on plant function, the correlation between traits and the relationship with environmental factors (Osnas et al., 2013; Westoby et al., 2013). In this study, variation in network parameters and node parameters was used to quantify differences between mass-based LTNs and area-based LTNs. Mass-based LTNs had a higher *edge density*, *diameter*, and shorter *average path length* than area-based LTNs. Therefore, leaf traits appear to be more strongly coordinated on a mass basis than on an area basis. This result supported those of previous studies (Wright et al., 2004). It might be attributed to the LMA-LL spectrum being related to mass-based nutrient concentrations (Wright et al., 2004).

### Leaf Lifespan and Leaf Nitrogen Are the Key Traits in Leaf Trait Networks

The environmental selection of functional traits with high centrality in LTNs may affect the whole phenotype. Consequently, it is necessary to identify the “key traits” in complex relationships among multiple traits. The “key traits” in LTNs might play important roles in regulating critical functioning or might be involved in regulating key functions, strongly influencing higher-level properties (e. g., fitness) (Koschützki and Schreiber, 2008). In this study, we showed that the *degree* of LL was the highest in mass-based LTNs; thus, LL was the “hub trait.” The cost of constructing carbon and carbon gain in leaves is directly related to LL (Reich et al., 1992, 1999), with LL likely being determined by LMA mechanistically. In other words, a higher LMA facilitates a higher LL owing to the higher carbon mass per area, or LL may also be associated with A_mass_. For instance, at the whole-plant scale, non-optimal resource use might arise if high-performance leaves lived long enough to experience self-shading from canopy growth (Ackerly and Bazzaz, 1995). Thus, the environmental selection of LL might strongly limit the variability of other leaf economic traits. We also found that N_mass_ had the highest *closeness* and *betweenness*; thus, N_mass_ acts like a bridge in LTNs, linking other leaf economic traits. This phenomenon might be attributed to nitrogen being allocated to cell walls and Rubisco (Onoda et al., 2017), mediating the trade-off between the structure and physiology of leaves, to some extent.

### Variation in Leaf Trait Networks Among Growth Forms and Life Forms

Variation in the network parameters of LTNs could quantify the interdependence of multiple traits. Compared with the LTNs of trees, the LTNs of shrubs had higher *edge density*, shorter *diameter*, and shorter *average path length*. All these network parameters suggest that the interdependence of leaf economic traits was higher in shrubs than in trees. A higher interdependence among traits might allow for the efficient acquisition and mobilization of resources (Flores-Moreno et al., 2019). Many studies have pointed out that plants with low resource availability likely face stronger selection and, thus, tend to have tighter trait correlations and trade-offs (Liu et al., 2019). For example, leaf economic traits and leaf hydraulic traits are decoupled in humid regions (Li et al., 2015), but are coupled in arid regions (Yin et al., 2018). Compared with trees, the availability of light resources could be limited for understory shrubs, and the availability of water resources could be limited for shrubs of open habitats. Consequently, shrubs adopt a cost-effective strategy that allows leaf economic traits to strongly correlate with each other, facilitating efficient functioning. The interdependence among leaf economic traits of broadleaved trees was higher than that of conifer trees. Therefore, compared with coniferous trees, the higher photosynthetic rate of broad-leaved trees may be the result of stronger interdependence of individual traits and multiple traits. Our results (except for leaf vein traits (Brodribb and Feild, 2010)) might provide novel evidence explaining why angiosperms, rather than gymnosperms, dominate the plant world.

### Future Directions and Challenges for Leaf Trait Networks

The network analysis is used to explore complex relationships among global leaf economic traits, and LTNs are established. The network parameters could help us identify key traits and quantify the interdependence of multiple traits. However, several hurdles challenge network optimization. Theoretically, the construction of plant trait network needs more matching trait data from different organs and different plant species. First, measuring and collecting many trait data from different organs is an important premise. Plant trait database TRY^3^ is one of the largest databases in the world, which can provide a strong database for the construction of trait networks (Kattge et al., 2011, 2020; Borgy et al., 2017). However, compared with the measured data, many interpolation data may cause large errors. For the construction of PTNs, it is best to use the same method for the measurement of traits as much as possible, even on the same plant (Westoby et al., 2002). Such networks could then be used to obtain an accurate representation of the ability of plants to adapt to various environments in the future. Second, this study only presents a typical example; however, more parameters must be studied and more quantitative methods with ecological significance must be developed.

Ultimately, the concept of trait networks could be applied to explore how trait networks of plants: (1) vary across climate zones and different regions; (2) vary along successional gradients, and (3) respond to disturbance and global climate change.

## Conclusion

Leaf trait networks provide an effective approach to explore how plants respond to the environment, with many promising applications.

## Data Availability Statement

The datasets presented in this study can be found in online repositories. The names of the repository/repositories and accession number(s) can be found below: https://www.try-db.org.

## Author Contributions

NH planned and designed the research. YL, CL, NH, LX, ML, and JZ analyzed the data and wrote the manuscript. All authors contributed to the article and approved the submitted version.

## Conflict of Interest

The authors declare that the research was conducted in the absence of any commercial or financial relationships that could be construed as a potential conflict of interest.

## Publisher’s Note

All claims expressed in this article are solely those of the authors and do not necessarily represent those of their affiliated organizations, or those of the publisher, the editors and the reviewers. Any product that may be evaluated in this article, or claim that may be made by its manufacturer, is not guaranteed or endorsed by the publisher.

## Footnotes

1
https://www.try-db.org

2
www.estimationstats.com

3
https://www.try-db.org
