# Supplementary material for: Leaf Trait Networks Based on Global Data: Representing Variation and Adaptation in Plants
Source: Front Plant Sci. 2021 Dec 7;12:710530. doi: 10.3389/fpls.2021.710530 (PMC8688851; doi:10.3389/fpls.2021.710530)
Supplement: Supplementary file 1 [file Data_Sheet_1.docx]

**Leaf trait networks based on global data: Representing variation and adaptation in plants**

Ying Li ^1,2^, Nianpeng He ^1,3,4*^(0000-0002-0458-5953), Congcong Liu ^1,3^ (0000-0003-3949-4194), Li Xu ^1,3^, Mingxu Li ^1,3^, Jiahui Zhang ^1,3^

^1^ Key Laboratory of Ecosystem Network Observation and Modeling, Institute of Geographic Sciences and Natural Resources Research, Chinese Academy of Sciences, Beijing, 100101, China

^2^ School of Ecology and Nature Conservation, Beijing Forestry University, Beijing 100083, China

^3^ College of Resources and Environment, University of Chinese Academy of Sciences, Beijing, 100049, China

^4^ Key Laboratory of Vegetation Ecology, Ministry of Education, Northeast Normal University, Changchun, China

*Correspondent author: N.H. (henp@igsnrr.ac.cn)

Institute of Geographic Sciences and Natural Resources Research, Chinese Academy of Sciences, Beijing 100101, China

Tel.: +86-10-64889263; Fax: +86-10-64889399

E-mail: [henp@igsnrr.ac.cn](mailto:henp@igsnrr.ac.cn)

**Table S2 The variation of six leaf traits among different plant categories.**

| **Plant categories** | | **Traits** | **n**^†^ | **mean** | **SD** |
| --- | --- | --- | --- | --- | --- |
| **all plants** | A_mass_^‡^ | 770 | 1.98 | 0.34 |  |
| **broad-leaf** | A_mass_ | 639 | 2.03 | 0.31 |  |
| **Needle** | A_mass_ | 59 | 1.51 | 0.25 |  |
| **shrubs** | A_mass_ | 234 | 1.9 | 0.28 |  |
| **trees** | A_mass_ | 352 | 1.87 | 0.3 |  |
| **all plants** | LL | 750 | 0.96 | 0.43 |  |
| **broad-leaf** | LL | 564 | 0.93 | 0.42 |  |
| **Needle** | LL | 53 | 1.47 | 0.34 |  |
| **shrubs** | LL | 226 | 1.04 | 0.38 |  |
| **trees** | LL | 363 | 1.1 | 0.38 |  |
| **all plants** | LMA | 2370 | 1.99 | 0.3 |  |
| **broad-leaf** | LMA | 1529 | 2 | 0.28 |  |
| **Needle** | LMA | 111 | 2.54 | 0.26 |  |
| **shrubs** | LMA | 733 | 2.14 | 0.32 |  |
| **trees** | LMA | 967 | 2.03 | 0.22 |  |
| **all plants** | N_mass_ | 2061 | 0.23 | 0.24 |  |
| **broad-leaf** | N_mass_ | 1420 | 0.23 | 0.24 |  |
| **Needle** | N_mass_ | 111 | -0.07 | 0.22 |  |
| **shrubs** | N_mass_ | 625 | 0.09 | 0.27 |  |
| **trees** | N_mass_ | 929 | 0.24 | 0.18 |  |
| **all plants** | P_mass_ | 752 | -1.1 | 0.39 |  |
| **broad-leaf** | P_mass_ | 522 | -1.1 | 0.39 |  |
| **Needle** | P_mass_ | 71 | -1.39 | 0.39 |  |
| **shrubs** | P_mass_ | 366 | -1.28 | 0.39 |  |
| **trees** | P_mass_ | 356 | -0.91 | 0.27 |  |
| **all plants** | Rd_mass_ | 274 | 0.99 | 0.27 |  |
| **broad-leaf** | Rd_mass_ | 231 | 1.02 | 0.26 |  |
| **Needle** | Rd_mass_ | 33 | 0.76 | 0.2 |  |
| **shrubs** | Rd_mass_ | 97 | 0.93 | 0.25 |  |
| **trees** | Rd_mass_ | 117 | 0.91 | 0.25 |  |

^†^n: the number of species in each plant categories; SD: standard deviation;

^‡^ A_mass_: photosynthetic assimilation rates; LL: leaf lifespan; LMA: leaf mass per area; N_mass_: leaf nitrogen; P_mass_: leaf phosphorus; R_mass_: dark respiration rate.

**Table** **S3 The results of principal components analyze for global leaf trait data**

| **Trait** | **PC1** | **PC2** | **PC3** | **PC4** | **PC5** | **PC6** |
| --- | --- | --- | --- | --- | --- | --- |
| **LL**^‡^ | –0.85 | 0.38 | –0.04 | 0.26 | 0.25 | 0.03 |
| **LMA** | –0.88 | –0.17 | 0.4 | 0.12 | –0.17 | 0.04 |
| **N_mass_** | 0.91 | 0.25 | 0.1 | 0.27 | –0.1 | –0.09 |
| **P_mass_** | 0.78 | 0.59 | 0.1 | –0.1 | –0.09 | 0.09 |
| **A_mass_** | 0.86 | –0.41 | –0.15 | 0.24 | –0.03 | 0.08 |
| **R_mass_** | 0.88 | –0.19 | 0.31 | –0.06 | 0.30 | 0 |

^†^ PC1, PC2, ……PC6 represented the score of principal components, respectively

^‡^ LL: leaf lifespan; LMA: leaf mass per area; N_mass_: leaf nitrogen; P_mass_: leaf phosphorus; A_mass_: photosynthetic assimilation rates; R_mass_: dark respiration rate.


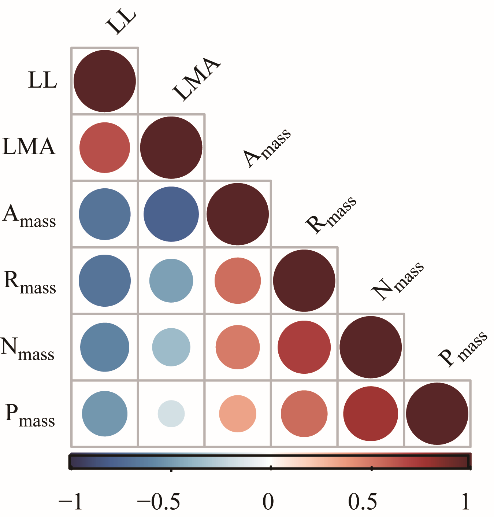


**Fig. S1 Bivariate relationships among six leaf traits on leaf mass.** Color from blue to red illustrated an increasing correlation strength. Blue and red circle showed negative and positive correlations, and the correlation strength was shown as the size of circle. LL: leaf lifespan; LMA: leaf mass per area; N_mass_: leaf nitrogen; P_mass_: leaf phosphorus; Amass: photosynthetic assimilation rates; R_mass_: dark respiration rate.


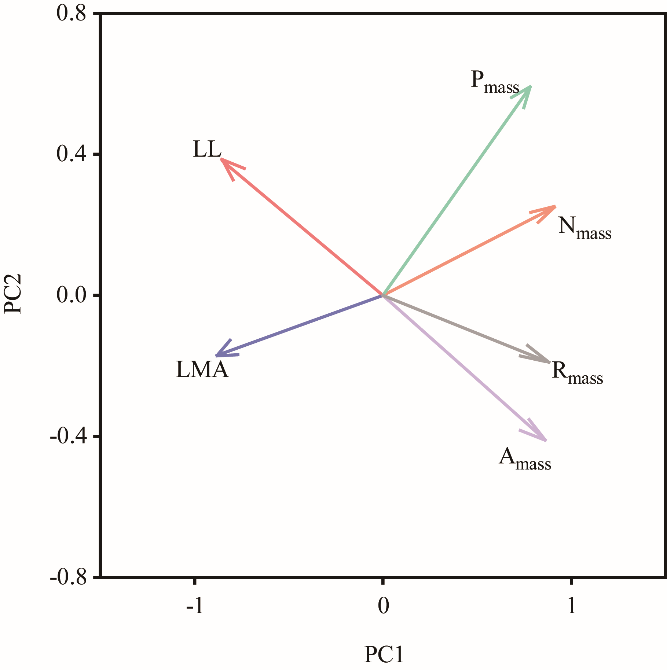


**Fig. S2 Principal components analyses of global leaf trait data on mass.** Arrow represented leaf traits, and the dotted line represented the distance between traits. It is impossible to draw the six-dimensional space, this figure therefore was drawn as the first and the second principal component. The distance between traits was the Euclidean distance of the six-dimensional space with 1-6 principal components as the coordinate axis. LL: leaf lifespan; LMA: leaf mass per area; N_mass_: leaf nitrogen; P_mass_: leaf phosphorus; A_mass_: photosynthetic assimilation rates; R_mass_: dark respiration rate.


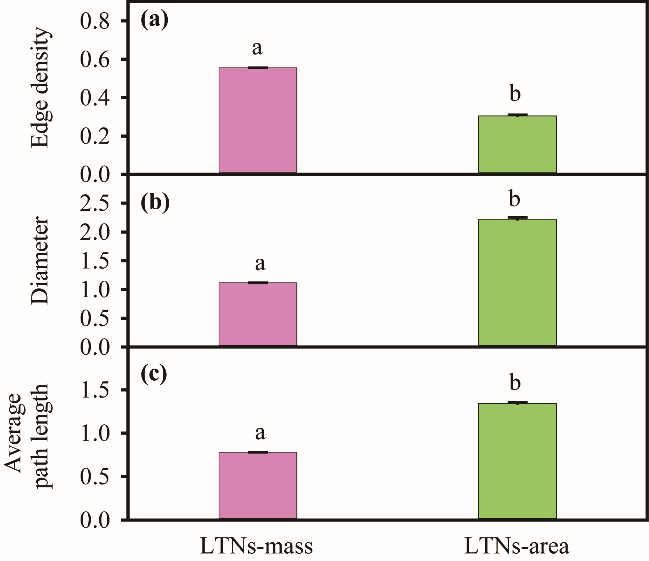


**Fig. S3 differences on the overall parameters between trait networks on mass-based and area-based leaf traits.** Different letters indicated the significant difference between two networks (*P* < 0.05). Error bars were represented standard error (SE).


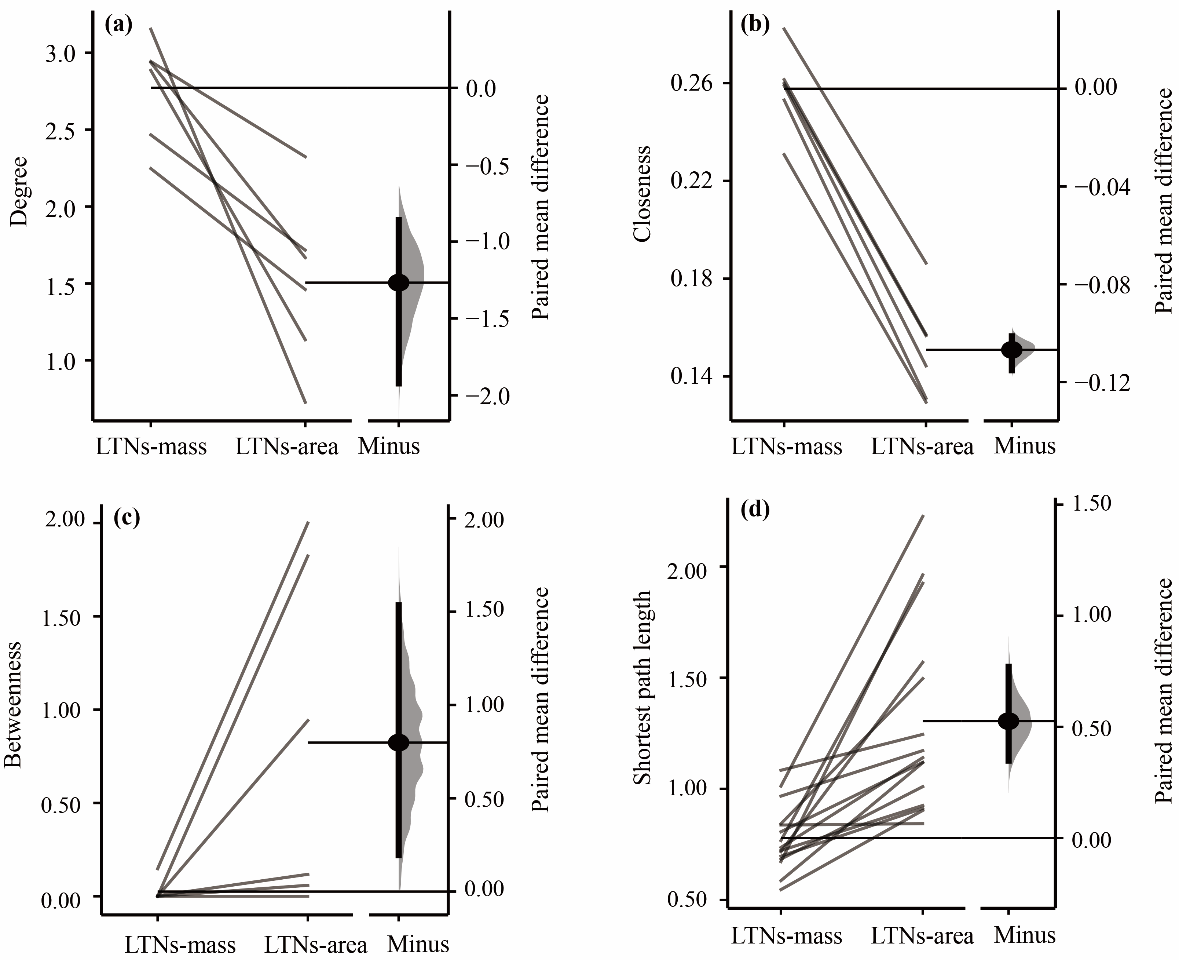


**Fig. S4 Cumming estimation plot for the paired mean difference for degree (a), closeness (b), betweenness (c) and distance (d) between trait networks on mass-based (LTNs-mass) and area-based leaf traits (LTNs-area).** The paired mean difference between LTNs-mass and LTNs-area was shown in the above plots. Both groups were plotted on the left axes as a slope graph. The paired mean difference was plotted on a floating axes on the right as a bootstrap sampling distribution. The mean difference was depicted as a dot, and the 95% confidence interval was given in the ends of the vertical error bar.


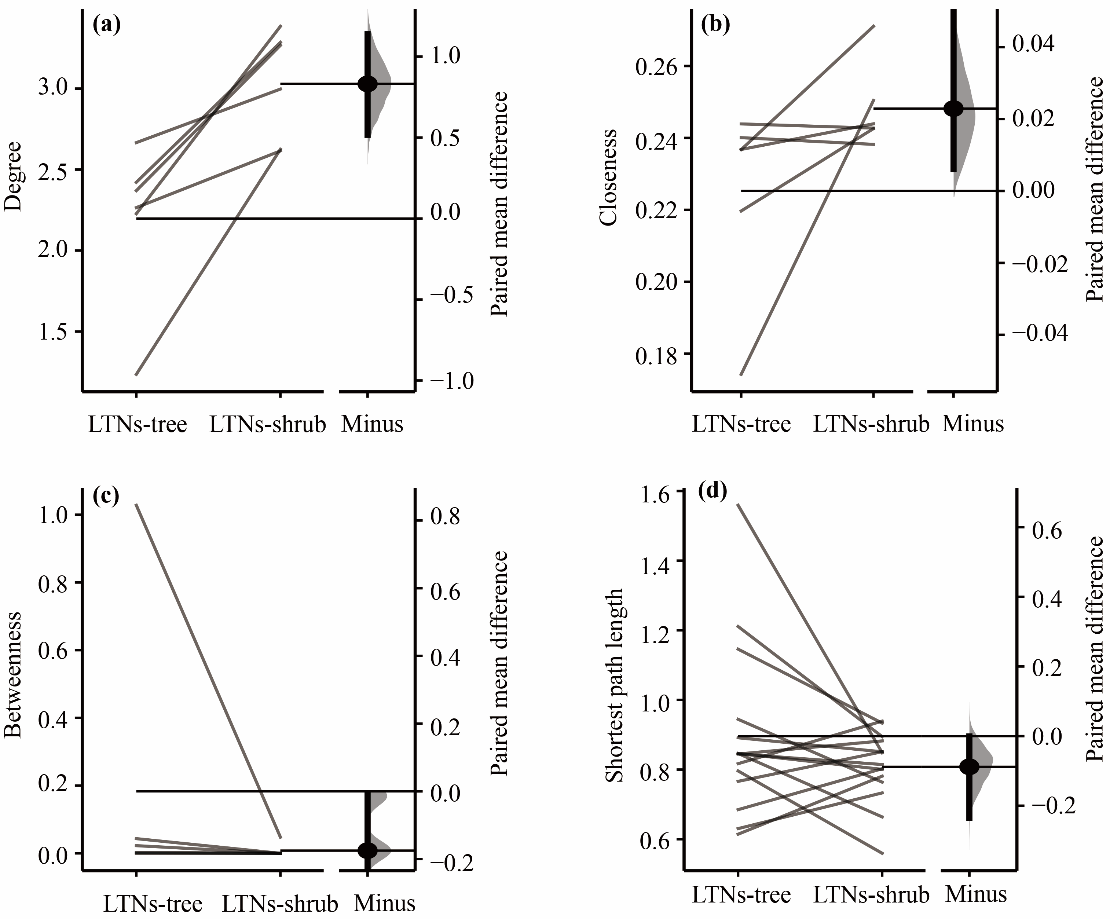


**Fig. S5 Cumming estimation plots for the paired mean difference degree (a), closeness (b), betweenness (c) and shortest path length (d) of LTNs among different plant growth forms.** The paired mean difference between trees and shrubs was shown as the above plots. Each paired set of observations was connected by a line. The paired mean difference was plotted on a floating axes on the right as a bootstrap sampling distribution. The mean difference was depicted as a dot; the 95% confidence interval is represented by the ends of the vertical error bar.


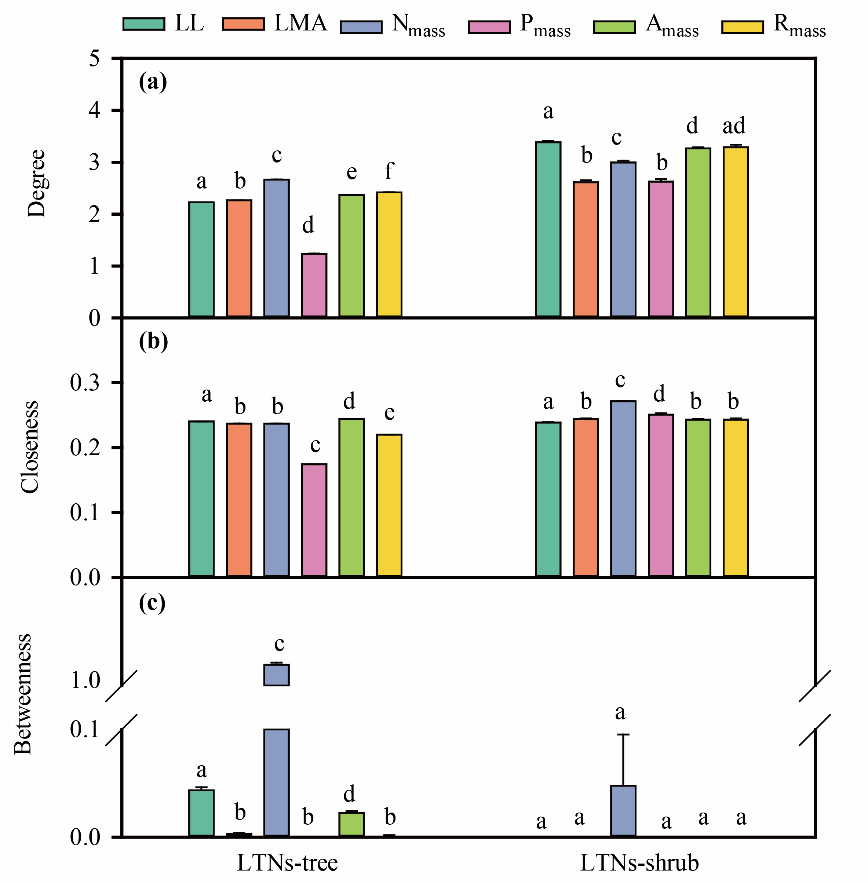


**Fig. S6 Variation in degree, closeness, and betweenness for different nodes (or traits) of leaf trait networks (LTNs) among different plant growth forms.**

Different letters show a significant difference among leaf traits (*P* < 0.05). Error bars represent standard error (SE).


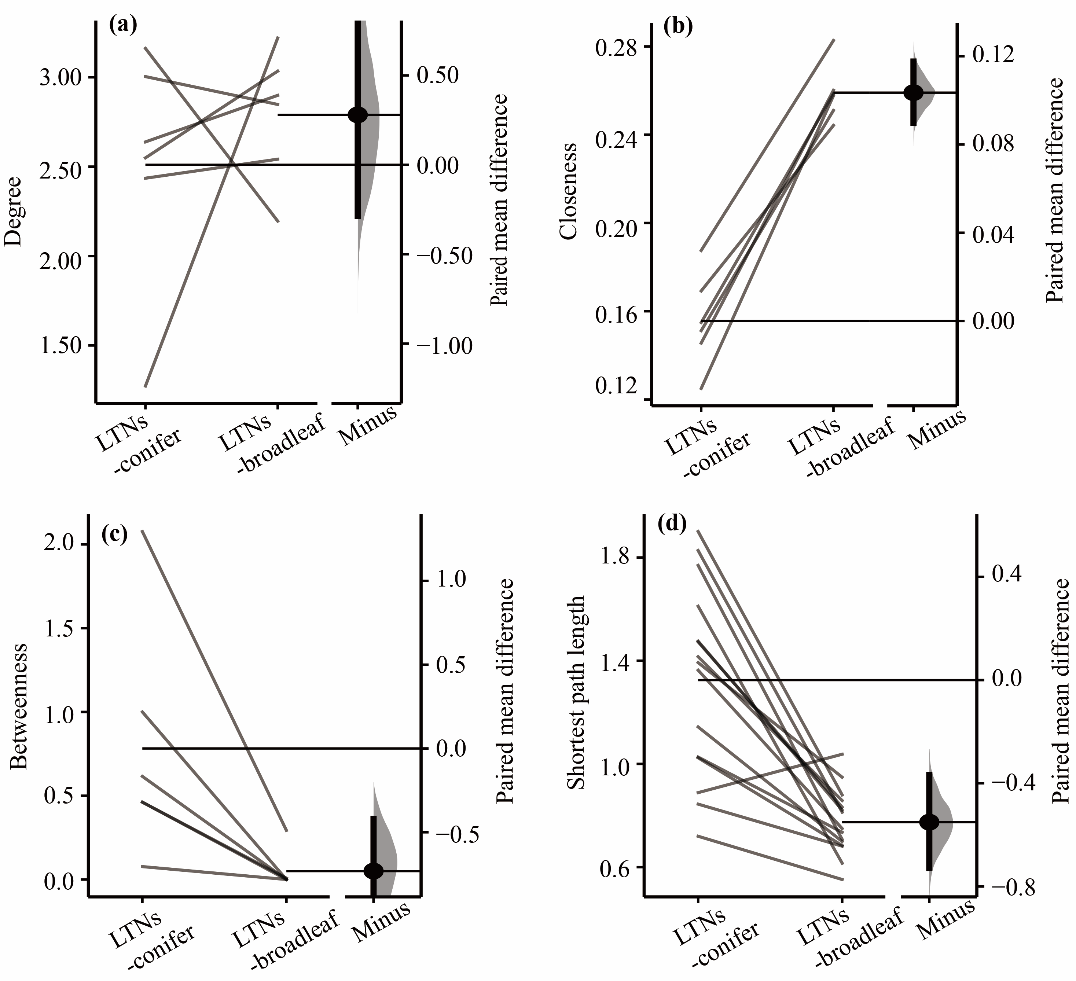


**Fig. S7 Cumming estimation plot for the paired mean difference for degree (a), closeness (b), betweenness (c) and shortest path length (d) of LTNs among different life forms.** The paired mean difference between coniferous and broad-leaved plants is shown in the above plots. Each paired set of observations was connected by a line. The paired mean difference is plotted on a floating axes on the right as a bootstrap sampling distribution. The mean difference was depicted as a dot; the 95% confidence interval is shown as the ends of the vertical error bar.


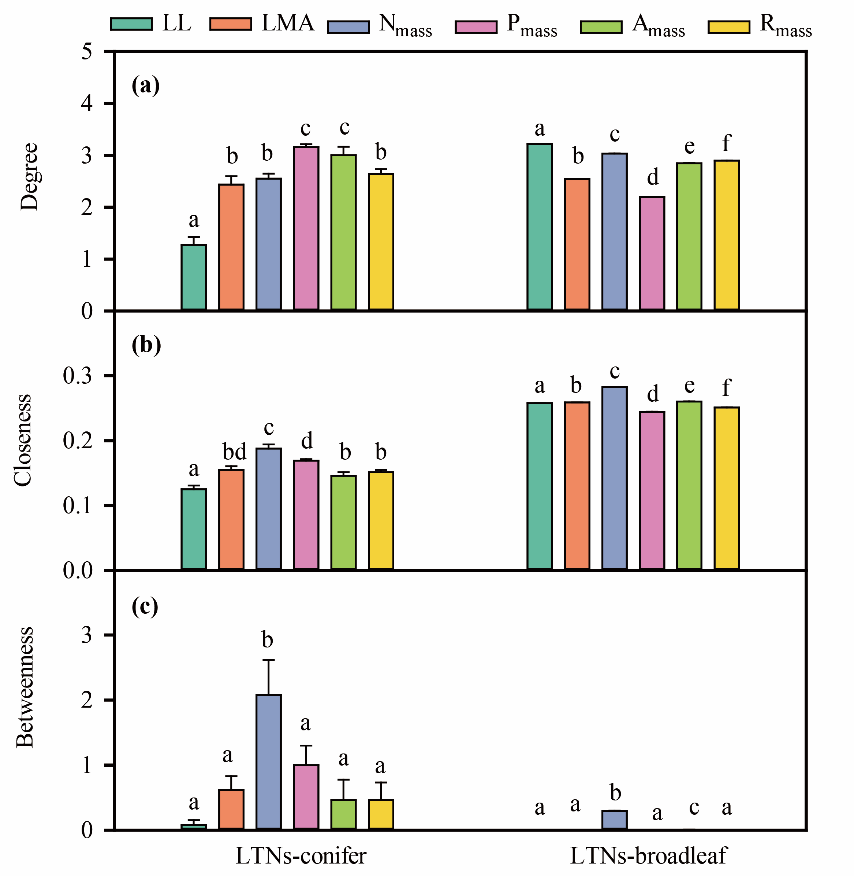


**Fig. S8 Variation in degree, closeness, and betweenness for different nodes (or traits) of leaf trait networks (LTNs) among different plant life forms.**

Different letters show a significant difference among leaf traits (*P* < 0.05). Error bars represent standard error (SE).
